# Molecular and Clinical Insights on the Complex Interaction between Oxidative Stress, Apoptosis, and Endobiota in the Pathogenesis of Endometriosis

**DOI:** 10.3390/diagnostics11081434

**Published:** 2021-08-09

**Authors:** Bogdan Doroftei, Ovidiu-Dumitru Ilie, Ioana-Miruna Balmus, Alin Ciobica, Radu Maftei, Ioana Scripcariu, Gabriela Simionescu, Delia Grab, Irina Stoian, Ciprian Ilea

**Affiliations:** 1 Faculty of Medicine, University of Medicine and Pharmacy “Grigore T. Popa”, University Street, no 16, 700115 Iasi, Romania; bogdandoroftei@gmail.com (B.D.); dr.radu.maftei@gmail.com (R.M.); isscripcariu@gmail.com (I.S.); gabi.ginecologie@gmail.com (G.S.); delianicolaiciuc@yahoo.com (D.G.); cilea1979@yahoo.com (C.I.); 2 Clinical Hospital of Obstetrics and Gynecology “Cuza Voda”, Cuza Voda Street, no 34, 700038 Iasi, Romania; 3 Origyn Fertility Center, Palace Street, no 3C, 700032 Iasi, Romania; 4 Department of Biology, Faculty of Biology, “Alexandru Ioan Cuza” University, Carol I Avenue, no 20A, 700505 Iasi, Romania; alin.ciobica@uaic.ro; 5 Department of Interdisciplinary Research in Science, “Alexandru Ioan Cuza” University, Carol I Avenue, no 11, 700107 Iasi, Romania; ioana.balmus@uaic.ro

**Keywords:** endometriosis, oxidative stress, apoptosis, vaginal microbiota, virome

## Abstract

Endometriosis (EMS) remains, to date, an intriguing and debilitating gynecological disorder that possesses a multifactorial substrate. Recent studies with the objective of elucidating its etiology highlighted the antagonistic effect of EMS on a multiple of processes involved in homeostasis. Although the current oxidative biomarkers clearly reveal the consequences induced by EMS, its implication in the associated inflammatory reactions could be much more complex. Besides the overproduction of reactive oxygen species (ROS) that leads to an exacerbated oxidative response, it also changes the normal expression of several pro-inflammatory modulators, reflected by the fluctuating activity of several pro- and anti-apoptotic mediators whose expression is impaired. In light of this topic, several studies elucidate the involvement of apoptosis in EMS, being brought controversial findings, even reports with no significant change. Further, some authors reported an abnormal expression of multiple genes that are crucial for the overall functionality of the female reproductive system. Cumulatively, it seems that the subsequent oxidative imbalance and apoptosis process impairment could further disrupt the normal removal of unnecessary biological products. Based on all gathered evidence, we could argue that the related stress state could determine human endobiota impairment, which could further participate in the inflammatory and main antioxidant enzyme changes occurring in EMS. Moreover, a correlation between endobiota integrity, inflammation, and oxidative stress (OS) was suggested in relation to the possible predisposition to pathogen determined infections.

## 1. Introduction

EMS is a common and enigmatic hormone-dependent gynecological disorder clinically described first in the 1920s. Even though more than one hundred years have passed since it was documented for the first time, its etiology still raises debate and controversy [1]. Clinically, EMS is defined as the presence of vascularized endometrial-like glandular tissue dispersed at ectopic sites and stroma outside the uterus. Despite the occurrence of these lesions within the peritoneal cavity (bladder, ovaries, colon, or peritoneum wall), the latest reports indicate the presence of such structures in the liver, lung, and brain as well [2,3].

Left untreated, EMS causes severe primary dysmenorrhea, chronic pelvic pain, dyspareunia, and infertility [4]. According to the latest issued figures, more than one hundred million women of reproductive age suffer from EMS (between 5–15% females worldwide). Approximately 90% report menstrual reflux, while 5–10% develop EMS [5].

It has been theorized to originate from endometrial fragments shed during menstruation. These lesions reach the peritoneal cavity via the Fallopian tubes, a process that is known as “retrograde menstruation” [6]. However, of the multitude of hypotheses that have been proposed in recent years—immune and stem cell dysfunction, embryonic rest, and coelomic metaplasia [7,8,9]—Sampson’s theory of retrograde menstruation remains the most solid argument of the origin of EMS to date [2,6,10,11].

Halme et al. suggested that most of the women display some degree of reflux of endometrial debris [12]. This argument is supported by the cumulative evidence according to which menstrual effluents could retrogradely scatter into the peritoneal cavity carrying viable endometrial cells [13,14,15,16,17].

Besides the standard clinical panel, EMS-diagnosed women are also at higher risk of developing autoimmune, cardiovascular, or gastrointestinal diseases and cancer [18]. From a mechanistical point of view, EMS could impair the apoptotic process, which could explain the abnormal cytokine storm and abnormal oxidative stress (OS) profile [19].

A disruption of the internal microenvironment could subsequently cause microbiome dysbiosis (e.g., pro-inflammatory cascades). With respect to the plethora of roles of the microbiome in various pathologies [20], endobiota could be the missing link. In this regard, Khan et al. 2018 discussed the new hypothesis known as “bacterial contamination” [21].

Considering the complex mechanisms underlying EMS development and the multiple hypotheses of pathological processes leading to EMS occurrence, we aimed to evaluate the possible relevance of OS, apoptosis, and dysbiosis in EMS. In this way, it is our goal to describe several mechanisms that can elucidate the outcomes and correlation of these particular processes.

## 2. Materials and Methods

For this work, literature searches were conducted independently using the main scientific databases (Web of Science, Scopus, Google Scholar, Science Direct, Cochrane Database for Systematic Reviews, PubMed/Medline). The initial information acquisition was based on database search using keywords such as “endometriosis”, “oxidative stress”, “apoptosis”, “endobiota”, “vaginal microbiota”, “cervical microbiota”, “gut microbiota”, “animal models”, and “human/patients” in all the relevant combinations. In the second step of selection, all the relevant literature up until June 2021 was categorized based on title, abstract content, and full content, and only the English written articles were further considered. Eight authors (O.-D.I., I.-M.B., R.M., I.S., G.S., D.G. and I.S.) independently inquired the information, with any differences in opinion being solved by common consent with the remaining three authors (B.D., A.C. and C.I.).

Based on all studies selected, in two tables (Table 1, Table 2 and Table 3), we present all main features that define each study.

## 3. Oxidative Stress and EMS

### Oxidative Stress and EMS

The implication of OS in EMS was previously described by several studies, which both addressed the screening of OS markers in EMS patients and mechanistical approaches in EMS animal models. In this way, the screening studies showed that several important oxidative markers are modified in EMS patients suggesting that OS could be an important component underlying and/or promoting EMS tissular damage. However, since correlation could not always imply causation, the way in which OS and EMS interact was not yet fully understood.

For instance, several studies showed that the typical OS markers changes suggest that EMS’s antioxidant defense could be impaired. Amreen et al., [22] reported that both superoxide dismutase (SOD) and glutathione peroxidase (GPx) antioxidant activities are significantly decreased in the blood and peritoneal fluid of EMS patients, while Turkyilmaz et al. [23] also found catalase (CAT) activity increased in the serum of 31 EMS-diagnosed women. Moreover, they showed that the pattern in which antioxidant enzymes activity varied is in direct correlation to EMS severity. Furthermore, it was demonstrated that non-enzymatic antioxidants, such as vitamin C, vitamin E, and native and total thiols, are significantly less present in the blood of EMS patients [23,24].

Regarding the effects of ROS production and accumulation in EMS, it was described that the decrease of antioxidant defense could further lead to significant molecular and tissular damage. It was shown that OS could have negative implications on proteins, lipids, and DNA [25]. While Alizadeh et al. [26] found no significant increase in lipid peroxidation (malondialdehyde—MDA) and protein peroxidation markers (carbonyls) in the blood of EMS patients, nor did Elsharkawy et al. [24] in the blood and follicular fluid (FF) of infertile EMS-diagnosed women, other studies bring relevant evidence of molecular damage following oxidative balance impairment. Thus, Nasiri et al. [27] recently evaluated lipid peroxides (LPO) and total antioxidant capacity (TAC) in the serum and FF of women with EMS and reported significantly increased LPO, concomitant with decreased TAC in both fluids, as compared to healthy control women.

However, a correlation between systemic and peritoneal oxidative biomarkers could not be established following Montoya-Estrada et al.’s [28] study in which increased levels of carbonyls and LPO were found in EMS patients peritoneal fluid, while other protein oxidation product (protein dityrosine) levels were increased in the blood, as compared to the control group. Despite the fact that no correlation was found between the hemoglobin content of the peritoneal fluid and carbonyls and LPO and between the OS markers from blood and peritoneal fluid, the study reported a significant increase of MDA levels in the latter, suggesting that, together with the ischemia-modified albumin levels increase, oxidative balance impairment could play a determinant role in EMS-related tissular damage and a possible correlation in the co-occurring pro-inflammatory processes. In this regard, it was shown that several inflammation-related molecules are also regulated dependently or independently of ROS production and accumulation. Thézénas et al. [29] identified amine oxidase 3 and vascular adhesion protein 1 (AOC3/VAP1), in particular alkenal reductase PTGR1 and methionine sulfoxide reductase, as candidates for altered ROS landscape in EMS and possibly influencing the systemic and/or local inflammatory response. Furthermore, they observed important molecular changes in the ectopic lesion (such as iron overload, increased ROS production, and lipid peroxidation) and noted that ROS-derived 4-hydroxy-2-nonenal (4-HNE) over-production led to monocytic interleukin IL-8 release.

The implication of iron in the OS modulation occurring in EMS was also previously suggested on several occasions. In this way, Alizadeh et al. [26] showed that serum iron levels in patients with EMS were significantly increased and suggested that this metal ions could be a potent promoter of OS in EMS. The accumulation of iron ions was also reported by Polak et al. [30] while studying the overall oxidative status of peritoneal fluid of EMS patients suggesting that iron metabolism could be disrupted in this disease. Furthermore, since iron could catalyze many ROS species production and was also implicated in cellular dysfunction, necrosis, and apoptosis, Chen, Hayashi, and co-authors [31,32] investigated the mitochondrial structure and function of endometrial stromal cells (ESCs) in ovarian endometriosis (OE) in relation to mitochondrial and iron metabolism. They found that in the ESCs, mitochondria generated more ROS, while SOD2 was highly expressed in the endometrium, suggesting an abnormal energetic metabolism of ectopic ESCs. SOD2 promoted two distinct processes: Cell proliferation and migration in OE. Furthermore, in an OE murine model, concomitant with the iron accumulation and proteins and DNA alterations due to OS, as evaluated through the levels of 4-NHE and 8-hydroxy-2′-deoxyguanosine (8-OHdG), specific lesions were observed in the intestine, pancreas, and peritoneal wall, and time-dependent ovarian fibrosis development. Further evaluation of fibrotic tissue development in EMS in correlation with OS occurrence was conducted by González-Foruria et al. [33] who described the possible mechanism in which OS may also be implicated in ADAM17/Notch signaling pathway modulation culminating with fibrosis development. They showed that in deep infiltrating endometriosis (DIE), a direct correlation was observed between ADAM17 protein levels and increased advanced oxidation protein products (AOPPs) in the ectopic lesions, as well as the fact that the expression of Notch was abnormal for type-I collagen, which suggested fibrosis development.

**Table 1:** Summary content regarding the outcomes in which we investigated the oxidative and potential biomarkers.

Subject	Number	Type of Sample	Biomarker of Interest	Reference
Female	18 EMS patients	Ectopic and eutopic samples	Novel OS biomarker-AOC3/VAP1, alkenal reductase PTGR1 and methionine sulfoxidereductase	[29]
Female	133 women; 40 ectopic and 73 eutopic	Ectopic ESCs	Ectopic ESC ROS ↑ SOD2 ↑	[31]
Female	55 EMS patients out of 64	Blood and peritoneal fluid	SOD ↓ GPx ↓	[22]
Female	28 EMS patients and 23 controls	Plasma and peritoneal fluid	Carbonyl ↑ LPO ↑	[28]
Female	121 EMS patients and 81 controls	Peritoneal fluid	AOPP ↑ ADAM17 ↑ Notch ↑	[33]
Female	110 EMS patients and 119 with benign ovarian cysts	Peritoneal fluid	Haemoglobin ↑ Iron ↑ TOS ↑ TAS ↓	[30]
Female	30 EMS patients and 30 controls	Follicular fluid and serum	Vitamin C ↓ Vitamin E ↓ SOD ↑ MDA ≠	[24]
Female	63 EMS patients	Follicular fluid and serum	LPO ↑TAC ↑	[27]
Female	31 EMS patients and 27 controls	Serum	Serum native thiols ↓ Total native thiols ↓ CAT ↑	[23]
Female	30 EMS patients and 40 controls	Serum	Iron ↑ MDA ≠ Carbonyl ≠	[26]
Murine model			Iron ↑ FSHR ↓	[32]

↑ increased. ↓ decreased. ≠ no significant change.

## 4. Apoptosis and EMS

Apoptosis is a distinctive form of programmed cell death that provides efficient elimination of senescent cells and tissue homeostasis during the menstrual cycle without eliciting inflammatory reactions. While EMS’s severity proved to be directly correlated with a fulminant production of ROS, this pathology also exerts antagonistic effects towards the functionality of apoptosis. Under normal circumstances, apoptosis usually prevents the migration and accumulation of ectopic and eutopic endometrial cells by destroying them before they have the opportunity to form necrotic tissue [34,35].

Several recent studies thoroughly described this correlation and explained the mechanisms in which endometrial cells apoptosis is modulated. On the one hand, it was shown that one of the most important apoptotic pathways in EMS endometrium is the Bcl-2 family-mediated mechanism. Moreover, in an EMS animal model study, Mulyantoro et al. [36] reported the effects of Bcl-2/Bax modulation on the abdominal and pelvic peritoneal tissues EMS implants concluding that the fate of the endometrial implants in the peritoneal tissues could be directly correlated to the Bcl-2/Bax expression. Moreover, Delbandi et al. [37] showed that there could be several differences in this mechanism’s modulation considering the origin of the EMS cells (ectopic versus eutopic). In this way, it seems that the anti-apoptotic signaling molecules (Bcl-2 and Bcl-xL) are expressed with a higher frequency in the ectopic EMS tissues suggesting that the apoptotic signals modulation could be implicated in the fate of the EMS cells. Furthermore, the study also reported the implication of lower caspase-3 pro-apoptotic activity (as an important component of Fas apoptotic pathway) in the eutopic EMS tissues implying that EMS development could be a matter of extrinsic and intrinsic apoptosis modulation. However, the authors suggested that the Fas apoptotic pathway could be implicated in the EMS preventive mechanism induced by immune activation, thus showing an important correlation between apoptosis modulation and inflammatory response.

In this context, Li et al. [38] showed that the upregulation of caspase-3 expression is concomitant with and correlated to the expression of the signal transducer and activator of transcription 1 (STAT1), which is also an important signal molecule in inflammatory response modulation. In addition, in a study of the potential beneficial effect of Kuntai Capsule (KTC) on apoptosis, Zhong et al. [39] showed that the modulation of caspase-8, caspase-9, caspase-3, and cytochrome could promote apoptosis in the EMS tissues. However, the authors pointed that the positive effect of Kuntai Capsule formulation could be effective only on the ectopic EMS tissues, clearly suggesting the differences between the mechanisms underlying ectopic and eutopic apoptosis. Moreover, a similar study pointed out that OS could also be implicated in this equation, since a correlation (yet not causation) could be established between the increased level of serum ROS and the decreased antioxidant enzymes activity (namely, SOD and CAT) and apoptosis rate, Bax, Bcl-2, and caspase-3 expression. Furthermore, the authors described the mechanism in which ROS could be implicated in the mitochondrial apoptotic JNK signaling pathway. We previously discussed the implication of this type of apoptosis in accordance with iron-mediated mitochondrial OS. Thus, it seems that there could be a close interaction between the OS mechanisms occurring as important effects of EMS tissues stimulation and development and the cell proliferation/apoptosis signaling. Caspase-3 was also previously described to be a key modulation point in the EMS apoptotic pathways [40].

On the other hand, Zhong et al. [39] also mentions the importance of traditional Chinese medicine known as the Kuntai Capsule on EMS pathogenesis by normalizing the expression of several pro-inflammatory and apoptotic mediators after three weeks of administration. Meldrum et al. [41] demonstrated that the growth of ectopic endometrium could be sensitive to hormone stimulation. It was previously shown that the impairments of estrogen-mediated cellular signaling could lead to important changes in TNF-α and IL-1β activities [42], thus suggesting that hormonal and inflammatory modulation could be correlated in EMS through the estrogen receptor (ER)β implication. Additionally, the escape of immune surveillance caused by ERβ could further interact with the apoptotic machinery and cytoplasmic inflammasome in the cytoplasm. Further studies showed that estrogen could promote Bcl-2 regulation and thymic stromal lymphopoietin (TSLP) secretion [43] and influence the steroid receptor coactivator (SRC-1) [44] (progesterone pathway). The way in which the latter is implicated in EMS was also correlated to apoptosis mechanisms (TNFα-mediated apoptosis) and inflammation (inflammatory signaling in endometriotic lesions), according to Han et al., 2012 [45] and Han et al., 2015 [42]. In this context, it was shown that p53 levels in ovarian endometriotic tissues are also correlated to the hormonal modulation (secretory phase versus proliferative phase) [46] and that depo-medroxyprogesterone acetate (DMPA), a progesterone derivate, could modulate cell proliferation and apoptosis in EMS women, but only in eutopic lesions cases [47].

However, Song et al. [48] recently showed that Bcl-2 modulation could also be implicated in cell proliferation and apoptosis in both eutopic and ectopic EMS tissues. In this way, the study that evaluated the EMS cell survival reported that the knockdown of YAP in ectopic endometrial stromal cells diminished cell proliferation but increased cell apoptosis concomitant with the decrease in expression of the transcriptional enhancer factor TEF-1 (TEAD1), connective tissue growth factor (CTGF), and Bcl-2. The overexpression of YAP also promoted an exaggerated proliferation and weakened apoptosis of normal endometrial stromal cells suggesting that the Hippo/Yes-associated protein (YAP) pathway is also an important component of apoptosis regulation in both ectopic and eutopic EMS lesion [48].

Other candidates for the potent apoptotic modulators in EMS were described by Bilgic et al. [49]. They showed that downregulation and/or alteration of endocannabinoid receptors could mediate the underlying mechanism of EMS and adenomyosis. Both CB1 and CB2 and also several key enzymes on the endocannabinoid pathway, such as fatty acid amide hydrolase (FAAH), N-acyl phosphatidylethanolamine phospholipase D (NAPE-PLD), diacylglycerol lipase (DAGL), and monoacylglycerol lipase (MAGL), were significantly impaired in endometriotic and adenomyotic tissues leading to a valid model of apoptotic signaling in EMS.

The much more complex TNF-α-mediated apoptosis in EMS still remains to be fully elucidated, since Tian et al. and Long et al. [50,51] described the mechanism through which the activation of this process could also be mediated by death-associated protein kinase 1 (DAPK1), c-Jun, and miRNAs. Another yet fully undiscovered mechanism is the implication of Ca+ and Cav1.3 channels in cell proliferation, aggregation, and apoptosis [52]. However, Yang et al. [52] already described the implication of the Cav1.3 channel, which is expressed in EMS tissue and primary ESCs cells and is highly sensitive to prostaglandin 2 and could modulate caspase 3-mediated apoptosis. The mitogen-activated protein kinase/ERK kinase (MEK)/extracellular signal-regulated kinase (ERK) signaling pathway has also been proposed to fulfil the important role(s) in cell proliferation, apoptosis, and migration. This is the reason why Chen et al. [53] studied these pathways in the EMS context and found that the modulation of these processes could lead to the proliferation, migration, and apoptosis control of the endometrial stromal cells in EMS.

**Table 2:** Summary content regarding the outcomes in which we investigated the apoptotic and potential markers.

Subject	Number	Regulatory Pathway	Reference
**Experimental Models**			
BALB/c mice	*n* = 33 mice	Bcl-2 ↓ Bax ↓	[36]
BALB/c nude	*n* = 5 per group	Bcl-2 ↑↓	[48]
C57BL/6J mice	*n* = 3 per group	ERβ ↑	[44]
Normal (C57BL/6J), ERβ^−/−^ (B6;129P2-Esr2^tm1Unc^/J), NALP3^−/−^ (B6.129S6-*Nlrp3^tm1Bhk^*/J), and SCID (NOD.CB17-*Prkdcscid*/J). ERBAI and ROSA^LSL:ERβ,^ ROSA^LSL:ERβ^:PR^Cre/+^being generated by crossing ROSA^LSL:ERβ^ with PR^Cre+^	*n = 4*	ERβ ↑↓	[42]
Normal C57BL/6J, *Tnf*^−/−^ (B6;129S-Tnf^tm1Gkl^/J), *Mmp9*^−/−^ (FVB.CgMmp9^tm1Tvu^/J), GFP-expressing (C57BL/6-Tg(CAG-GFP)10sb/J) and SCID (NOD.Cg-Prkfc^scid^B2mt^m1Unc^Il2rg^tm1Wjl^/Szj), SRC-1 null–was generated by crossing SRC-1-null with GFP-expressing	*n* = 6	MMP9 ↑	[45]
Sprague- Dawley rats	*n* = 74	caspase-3 ↑ caspase-8 ↑ caspase-9 ↑ cytochrome ↑	[39]
**Culture(s)-In Vivo**			
Human endometrial stromal cells	*n* = 32; *n* = 16 peritoneal or OE or both and *n* = 16 controls; *n* = 12 dermoid cysts and *n* = 3 serous cystadenoma and *n* = 1 simple ovarian cyst	caspase-3 ↑ miR-21–5p ↑	[40]
Human endometrial stromal cells	*n* = 25 OE and *n* = 5 pelvic EMS	Bcl-2 ↑↓	[43]
Human ectopic and normal endometrial cells		STAT1 ↑ caspase-3 ↑	[38]
Human eutopic and ectopic ESCs controls	*n* = 17, *n* = 11, and *n* = 15	Bcl-2 ↑ Bcl-xL ↑ caspase-3 ↓ VEGF-A ↑ HGF ↑	[37]
**Cell line(s)-In Vitro**			
Female/CRL-7566 cell line	*n* = 20 EMS patients, *n* = 17 adenomyosis and *n* = 19 controls; *n* = 12 proliferative, *n* = 7 secretory; Endometriosis tissues were divided as: *n* = 9 cystic and *n* = 11 non-cystic	CB1 ↓ CB2 ↓	[49]
Female/HEK293T, CRL7566, CRL-11731 cell lines	*n* = 10 OE, *n* = 10 ovarian cancer, and *n* = 10 controls	miR-191 ↑↓	[50]
Female/CRL-7566 cell line	*n* = 20 eutopic and ectopic endometrium	miR-29c ↑↓ c-Jun ↑↓	[51]
Female/hEM15A cell line	*n* = 30 OE and *n* = 30 controls	Cav1.3 ↑↓	[52]
**Human patients**			
Female	*n* = 30 OE and *n* = 29 controls	p53 ↑ p16 ↑ MDM2 ↓	[46]
Female	*n* = 28 EMS patients, *n* = 14 laparoscopy after DMPA injection and *n* = 14 laparoscopy without DMPA injection	PCNA ≠	[47]
Female	*n* = 30 EMS patients and *n* = 15 controls	Raf-1 ↑	[53]

↑ increased. ↓ decreased. ↑↓ regulatory. ≠ no significant change.

## 5. Endobiota and EMS

Regarding the microbiota interaction with the reproductive system functions, it was previously shown that the vaginal, cervical, and gut microbiota could be implicated in the pathogenesis of EMS. In this way, Ata et al. [54], in a study on the endobiota status in EMS-diagnosed women, showed the total absence of *Atopobium* in both vaginal and cervical microbiota of EMS group, and also the presence of *Gardnerella, Streptococcus, Escherichia, Shigella*, and *Ureaplasma* in the cervical samples. Moreover, Cregger et al. [55] suggested that the richness and phylogenetic diversity were increased in stage III EMS, as compared to healthy individuals, and that the surgical intervention for EMS tissues neutralization reversed the endobiota characteristics for a limited period of time. In this context, while the bacterial contamination EMS development hypothesis suggests that EMS pathogenesis and inflammation are correlated by the microbial pathogens reaching the endometrial space [56], it was shown that in an EMS mice model, the reduction of the ectopic lesions and the magnitude of the inflammatory response could both be modulated at the same time through antibiotic administration [57]. Furthermore, it is important to mention that both OS-related apoptotic signals and the inflammatory response are modulated differently, considering the origin of the elicitors. In this way, Chen et al. [58] suggested that, during vaginal inflammation due to pathogens invasion, the immune response could also be triggered by ROS overproduction, eventually leading to an apoptotic signal via neutrophilic activation (caspase-3 mediated). A similar mechanism was previously described for apoptotic modulation in the ectopic endothelial stromal cells underlying EMS tumors.

However, despite that the most prevalent microbiota component was the *Lactobacillus* genus members, Wei et al. [59] showed that as advancing up the reproductive tract, the microflora diversity increases, as they observed by sampling the vagina, posterior vaginal fornix, cervical mucus, endometrium, and peritoneal fluid. Moreover, Riganelli et al. [60] suggested that reproductive system-colonizing *Lactobacillus* species could play an important role in more than mucosa maintenance, as they observed that the differences between in vitro fertilized pregnant and non-pregnant women mainly reside in the vaginal and endometrial microbiota diversity. Moreover, it is known that lactobacilli are a continuous source of H_2_O_2_, which could contribute to intrinsic protective mechanisms, thus the possible implication of this microbiota component in OS and apoptosis modulation [59].

Furthermore, it could be suggested that there could be an interaction between the hormonal fluctuations during the menstrual cycle and reproductive system microbiota. However, disregarding that microbial taxa and potential functions proved to be positively correlated with the menstrual cycle or are over-represented in patients with adenomyosis and/or infertility caused by EMS [61], Akiyama et al. [62] showed that the distribution of microbiota in the cervical mucus of women with and without EMS is very similar and that regardless of the menstrual cycle phase, *Lactobacillus spp*. were dominant, concomitantly with an increased ratio of *Corynebacterium, Enterobacteriaceae, Flavobacterium, Pseudomonas,* and *Streptococcus* in the EMS group. *Enterobacteriaceae* and *Streptococcus* were considered to be significant candidates in the EMS group compared to in controls. Furthermore, considering the previously mentioned implications of estrogen in apoptosis, Leonardi et al. [56] thoroughly discussed the role of microbiota in estrogen metabolism in EMS with regards to the changes occurring in the gut microbiota. In this way, it was suggested that EMS development and progression could be very much influenced by the prevalence of the bacteria that produce enzymes for estrogen deconjugation in the gut.

Considering these aspects, further investigations were carried out on animal models in order to elucidate the way in which gut, vaginal, and endometrial microbiota are implicated in the EMS occurrence and development. In this way, using transgenic green fluorescent protein (GFP)^+^ donor mice and the standard antitumor and immunologic studies mice strain C57BL/6, Hantschel et al. [63] demonstrated that the uterine tissues fragment transplantation receiver mice exhibited significant differences in terms of gut microbiota, alongside the typical tissue morphology of the lesions and ease of differentiating GFP-host tissues. In a similar study, Yuan et al. [64] also described the gut microbiota alterations during EMS development via intraperitoneal injection of endometrial tissues in C57BL/6 mice, as both EMS and mock groups shared an identical microenvironment and richness until day 42. There was a significantly different ratio among *Firmicutes/Bacteroidetes* in the EMS group, and *Bifidobacterium* was also increased in this group, suggesting that lesion development and dysbacteriosis could be correlated. Considering this, Hu et al. [65] further analyzed the existence of an inflammatory response of the uterus in FMT-mice and observed that EMS induced by pathogenic bacteria inoculation could be more severe, but reversible. Moreover, considering the molecular changes that can be observed in this context, the authors noted a significant decrease in the short-chain fatty acids (SCFAs) in the feces of mice with dysbiosis and the administered treatment with sodium butyrate/propionate increased the overall concentration in both circulation and uterine tissues. Furthermore, the study reported the increase in pathogen load in the uteri of dysbiosis-associated mice, which could explain the restricted phagocytic ability and responsiveness of neutrophils.

A crucial yet underestimated microbial composition modulator could also be the pathogens encountered in the reproductive system. In this way, several studies reported high human papillomavirus prevalence [66,67,68] in association with EMS. In all these cases, the main aim was to investigate whether sexually transmitted viruses (STDs) or prokaryotes such as human HPVs, herpes viruses, and *Chlamydia trachomatis* in the lower and upper female genital tract are correlated with EMS injuries. Considering that herpes viruses and *Chlamydia trachomatis* were not detected in endometriosis lesions, HPV was prevalent in all samples analyzed in both categories: With and without EMS. The risk is higher, even double, in patients with EMS, with all positive patients having been previously detected with an HPV infection. As expected, the risk towards medium (md) and high (hr) HPV was in females with EMS in both lower and upper genital tract. However, Vestergaard et al. [69] contradicts these findings and sustain that no significant differences were observed in terms of virus prevalence in endometriotic lesions.

**Table 3:** Summary content regarding the outcomes in which we investigated the microbial taxa in EMS.

Subject	Number	Region Analyzed	Microbial Taxa Differences	Reference
Female	14 stage 3/4 ¾ EMS women and 14 controls	V3-V4 region 16S rRNA	Atopobium absence in the vaginal and cervical microbiota ↑ *Gardnerella* ↑ *Streptococcus* ↑ *Escherichia* ↑ *Shigella* ↑ *Ureoplasma* Prevalence of *Shigella* and *Escherichia* in the stool	[54]
Female	19 women; *n* = 10 distinct EMS stages and *n* = 9 controls	V3-V5 region 16S rRNA	Significant alterations among bacterial communities depending on the site from which the sample was collected and diagnosis	[55]
Female	36 EMS women and 14 controls	V4-V5 region 16S rRNA	Prevalence of Lactobacillus in the lower third of the vagina and posterior vaginal fornix. Differences among communities were visible starting from the cervical mucus of EMS patients, gradually increasing towards the reproductive system. ↑ Operational Taxonomic Units in the cervical mucus, endometrium, and peritoneal fluid.	[59]
Female	34 women	V4-V4 region 16S rRNA	Lactobacilli-dominant microenvironment in contrast to non-pregnant women, whereas the endometrial bacterial colonization was marker exclusively by a polymicrobial habitat in which lactobacilli were predominant	[60]
Female	39 EMS women and 30 controls	V5-V6 region 16S rRNA	Similar microbial taxa between women with and without EMS. ↑ Lactobacilli spp ↑ Corynebacterium ↑ Enterobacteriaceae in EMS group than in control (*p* < 0.05) ↑ Flavobacterium ↑ Pseudomonas ↑ Streptococcus in EMS group than in Control (*p* < 0.05)	[62]
Mice	C57BL/6 wild-type mice and transgenic C57BL/6-TgN(ACTB-EGFP)1Osb/J donor mice; 4 per cage-2 EMS and 2 sham-transplanted controls	V4-V5 region16S rRNA	No significant difference between groups, being speculated that EMS does not induce a dysbiosis during the acute phase of lesion formation.	[63]
Mice	C57BL6; 22 from which in 4 distinct intervals (day 7, 14, and 28) fecal sample was collected and the mice were sacrificed for model confirmation; 8 out of 10 in day 42.	V4 region 16S rRNA	Similar diversity and richness between EMS and mock mice. ↑ Firmicutes/Bacteroidetes ratio in EMS after 42 days ↑ Bifidobacterium in EMS group	[64]
Mice	BALB/c mice	V4 region 16S rRNA	↑ pathogen load in the uteri of gut microbiota-dysbiosis mice	[65]

↑ increased.

## 6. Conclusions

Based on all aspects discussed throughout this manuscript, we could suggest that endometriosis exerts a pronounced detrimental effect on an organism’s integrity. Overall, clear changes of oxidative biomarkers levels could be observed, the associated repercussions being branched and reflected by an abnormal expression of apoptosis-related mediators that ultimately lead to a dysbiosis. The current knowledge in endometriosis pathophysiology offers some explanation for the crucial roles that oxidative stress, apoptosis, and endobiota possess in various gynecological disorders, further outlining the multifactorial character of endometriosis.

## Data Availability

The datasets used and analyzed during the current study are available from the corresponding author on reasonable request.
